# Supplementary material for: Duodenases are a small subfamily of ruminant intestinal serine proteases that have undergone a remarkable diversification in cleavage specificity
Source: PLoS One. 2021 May 28;16(5):e0252624. doi: 10.1371/journal.pone.0252624 (PMC8162674; doi:10.1371/journal.pone.0252624)
Supplement: S1 Fig — We here show the loci of three additional lysozyme c related genes, LysL1, LysL4-Like and Lyz-Like6 for a comparison to show that gene duplications primarily have occurred in the lysozyme c locus as shown in Fig 1. (DOCX) [file pone.0252624.s001.docx]

# Supplementary Figure 1

**Analysis of the chromosomal loci for several additional lysozyme c related genes.** We here show the loci of three additional lysozyme c related genes, LysL1, LysL4-Like and Lyz-Like6 for a comparison to show that gene duplications primarily have occurred in the lysozyme c locus as shown in figure 1.

**
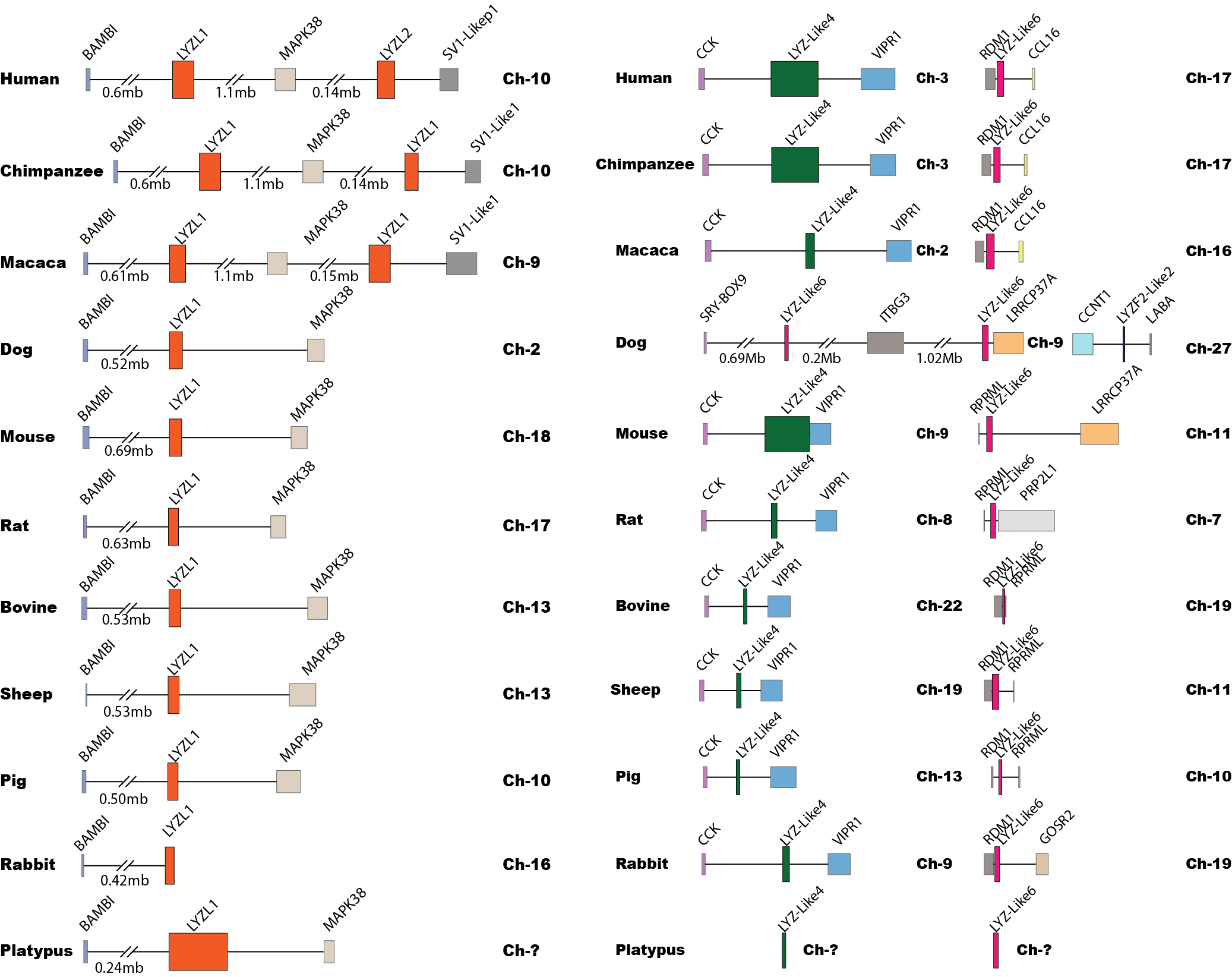
**
