# Supplementary material for: Duodenases are a small subfamily of ruminant intestinal serine proteases that have undergone a remarkable diversification in cleavage specificity
Source: PLoS One. 2021 May 28;16(5):e0252624. doi: 10.1371/journal.pone.0252624 (PMC8162674; doi:10.1371/journal.pone.0252624)
Supplement: S1 Table — (DOCX) [file pone.0252624.s002.docx]

**Table S1**

| Protein | NCBI accession No. | Motif | P1 position |
| --- | --- | --- | --- |
| **Protocadherin Fat 4 isoform X1** | XP_005217662.1 | QAFDK**D**SG | 1024 |
|  |  | HQFDR**E**SL | 1164 |
|  |  | SAFDS**D**SV | 3434 |
| **Collagen alpha-1(VII) chain** | XP_002697097.2 | TEFDL**D**AL | 95 |
|  |  | TAFDL**D**DV | 562 |
|  |  | SSYDL**D**GL | 921 |
| **FRAS1-related extracellular matrix protein 2 isoform X1** | XP_002691845.2 | IRFDK**D**ER | 1955 |
|  |  | SLFEE**E**ET | 1974 |
| **Protocadherin Fat 3 isoform X1** | XP_024842993.1 | IRFEK**D**AY | 378 |
|  |  | GRFEI**D**KA | 985 |
|  |  | VPFDR**E**EQ | 2044 |
|  |  | STWDR**D**TS | 2951 |
|  |  | GGYDV**D**SE | 4431 |
| **Protocadherin Fat 2 precursor** | NP_001179501.2 | GCFDI**E**LE | 762 |
|  |  | DAWDL**D**SS | 1161 |
|  |  | MDFDI**E**KT | 1392 |
|  |  | LQFDR**D**VY | 1967 |
|  |  | VGFDR**E**QQ | 2027 |
|  |  | HIYEAELA | 2485 |
|  |  | LAYDADEG | 2609 |
| **Laminin subunit beta-1 precursor** | NP_001193448.1 | FAYDC**E**SS | 171 |
|  |  | YIYEA**E**EA | 560 |
|  |  | MDFDR**D**VL | 1450 |
| **Protocadherin Fat 4 isoform X1** | XP_005217662.1 | QAFDK**D**SG | 1024 |
|  |  | FYFEE**E**QR | 1112 |
|  |  | HQFDR**E**SL | 1164 |
|  |  | SAFDS**D**SV | 3434 |
|  |  | EHYDI**D**NA | 4624 |
|  |  | MEYDR**E**KP | 4859 |
| **Mucin-5B** | XP_024843362.1 | EWFDV**D**YP | 838 |
|  |  | EWFDV**D**YP | 1298 |
|  |  | EWFDV**D**YP | 2451 |
|  |  | EWFDV**D**YP | 3062 |
|  |  | EWFDV**D**FP | 3595 |
| **Protocadherin Fat 1 isoform X1** | XP_024841984.1 | GNFDV**D**KL | 977 |
|  |  | EAFDP**D**SS | 1167 |
|  |  | SHFDV**D**RG | 1405 |
|  |  | RAYDA**D**SS | 2418 |
|  |  | PSWDF**D**YD | 4331 |
|  |  | GGYDI**E**SD | 4464 |
| **Cortactin-binding protein 2** | XP_005205505.2 | KEFDV**D**TL | 34 |
|  |  | SEFDT**E**RE | 237 |
|  |  | DDFEC**E**ST | 984 |
|  |  | LRWDG**E**PM | 1267 |
| **Laminin subunit gamma-2 isoform X1** | XP_002694254.1 | CIFDQ**E**LH | 42 |
|  |  | TFYEV**E**NI | 947 |
|  |  | REFDV**D**MD | 1068 |
| **Cadherin-16 precursor** | NP_001015550.1 | GPFDV**D**TE | 283 |
|  |  | RAFEL**D**ST | 394 |
|  |  | LDWEP**D**SA | 506 |
| **Maltase-glucoamylase, intestinal isoform X1** | XP_015326291.1 | VWYDY**E**TG | 762 |
|  |  | SSLDW**D**SQ | 1356 |
|  |  | VTWDV**D**SQ | 1637 |
| **Cadherin-23 precursor** | NP_001178135.2 | RELDY**E**TT | 196 |
|  |  | IPLDY**E**TV | 422 |
|  |  | FSLDK**D**TG | 511 |
|  |  | ARLDY**E**LI | 524 |
|  |  | RPLDY**E**QI | 631 |
|  |  | SLLDR**E**TK | 737 |
|  |  | AELDR**E**RI | 956 |
|  |  | VNLDR**E**TT | 1061 |
|  |  | DYLDY**E**TK | 1274 |
| **Collagen alpha-3(VI) chain isoform X1** | XP_024846030.1 | LDL**D**Y**E**LA | 2013,2015 |
|  |  | DGLDG**E**DG | 2116 |
| **Desmoplakin** | NP_001179297.1 | VCLDL**D**KV | 812 |
|  |  | PLYDL**D**LG | 862 |
|  |  | RLWDL**E**KQ | 892 |
|  |  | AIFDT**E**NL | 2642 |
