# Supplementary material for: Duodenases are a small subfamily of ruminant intestinal serine proteases that have undergone a remarkable diversification in cleavage specificity
Source: PLoS One. 2021 May 28;16(5):e0252624. doi: 10.1371/journal.pone.0252624 (PMC8162674; doi:10.1371/journal.pone.0252624)
Supplement: S1 Raw images — (PDF) [file pone.0252624.s003.pdf]

S1 raw images

All images in this PDF shows 4-12% pre-cast SDS-PAGE gels from Invitrogen (Carlsbad, CA, USA) stained with Coomassie brilliant blue and scanned with a Cannon flat bed scanner and saved as JPG files. The lanes marked with large X is not part of the final figures (4, 7 and 8).
The gels in figures 7 -12 are all showing the panels they appear in in the final figure. In the more complex figure 4 we mark all lanes in the individual panels with the corresponding names in the final figure for more easy identification.

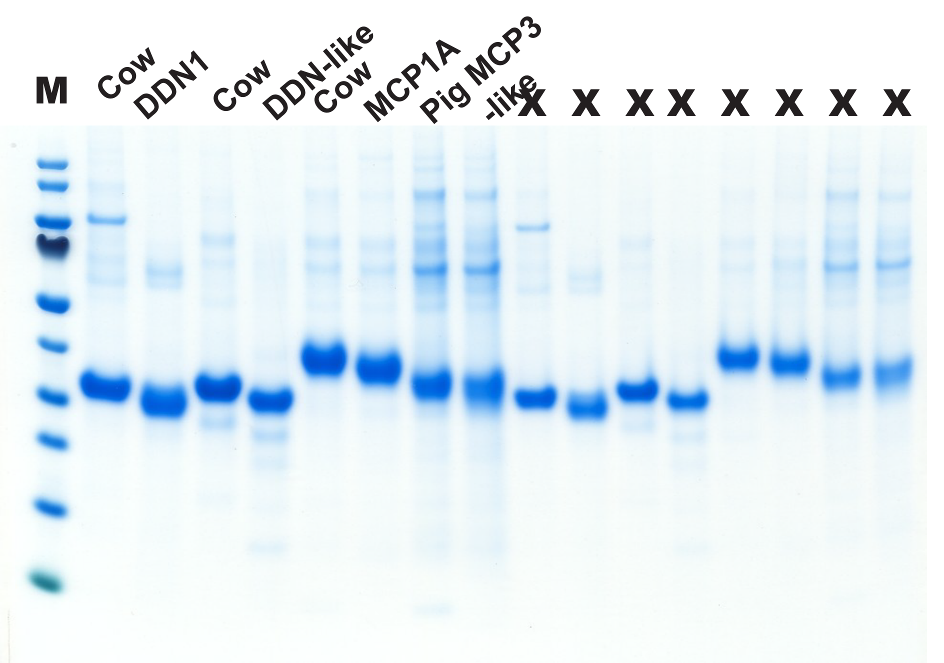

Figure 4

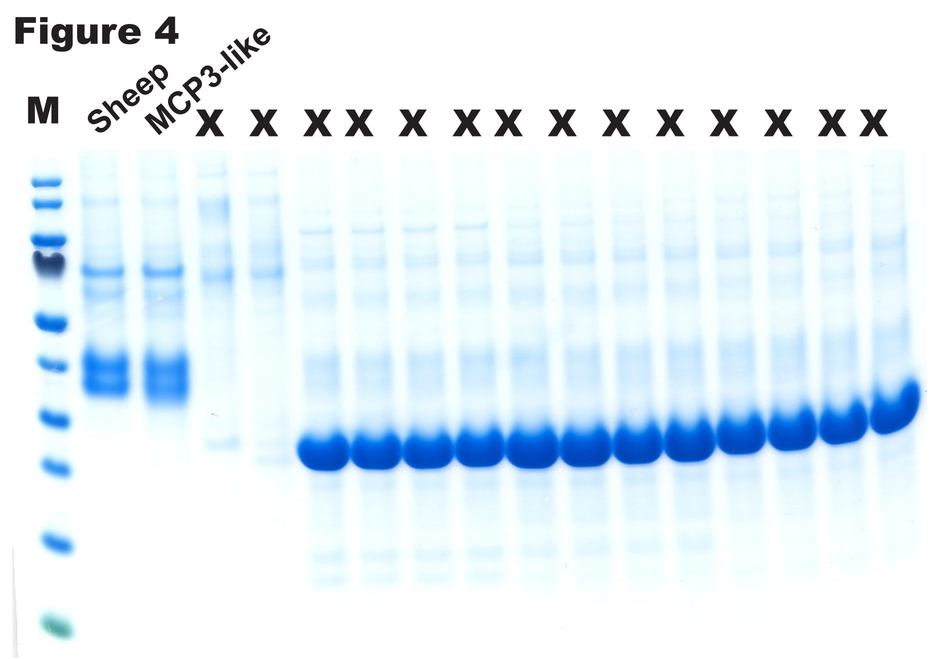

Figure 4

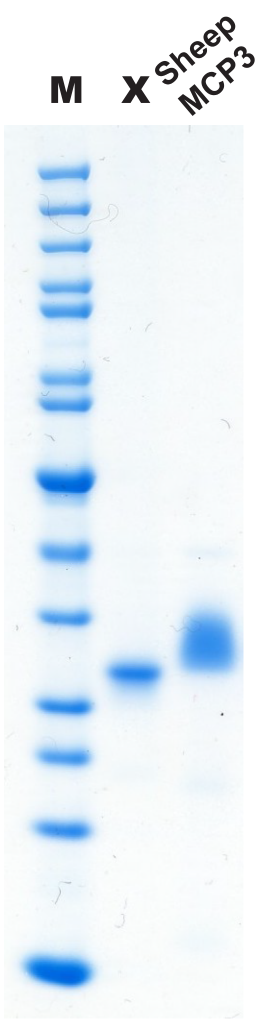

Figure 4

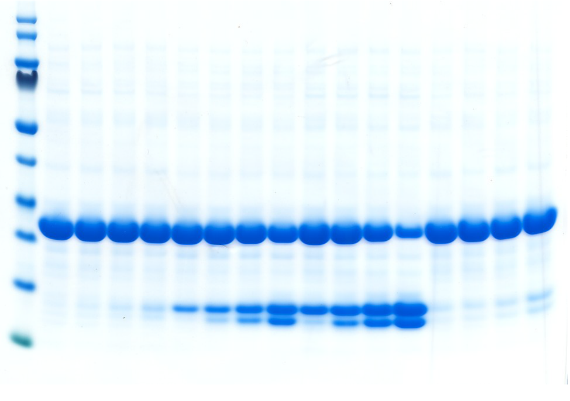

Figure 7C

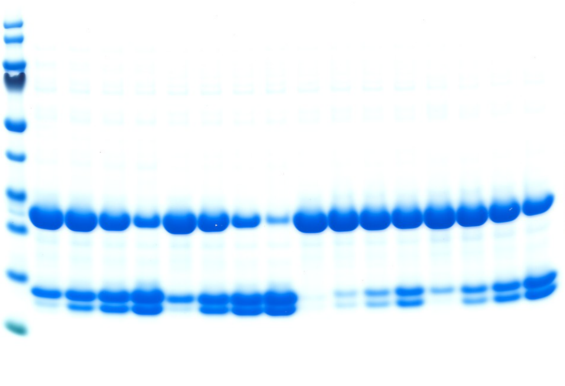

Figure 7D

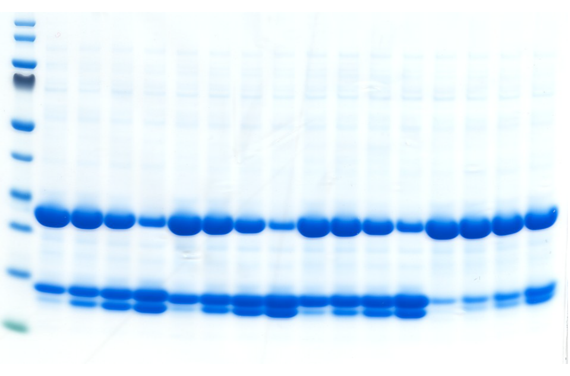

Figure 7E

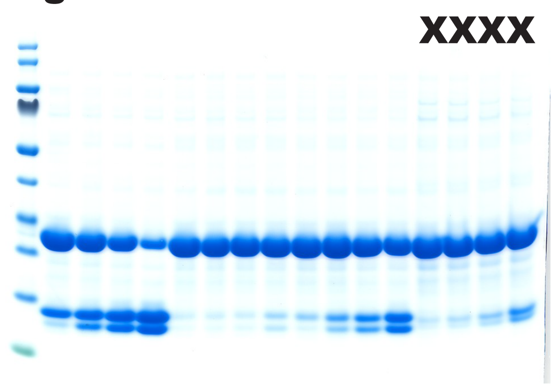

Figure 7F

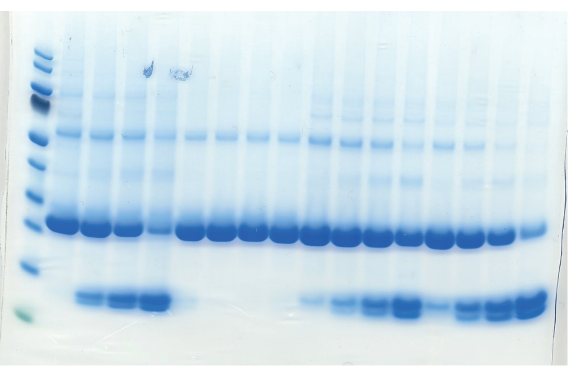

Figure 7G

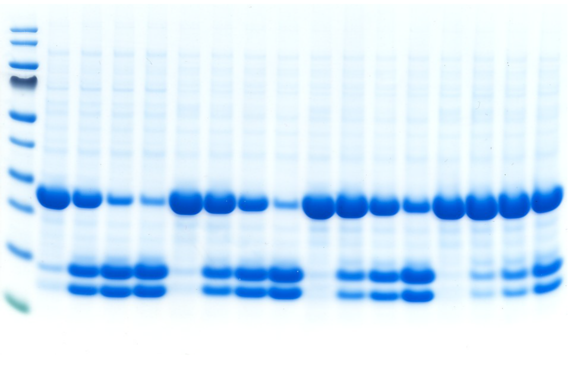

Figure 8A

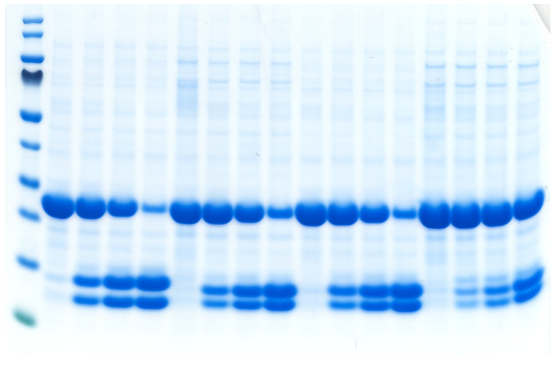

Figure 8B

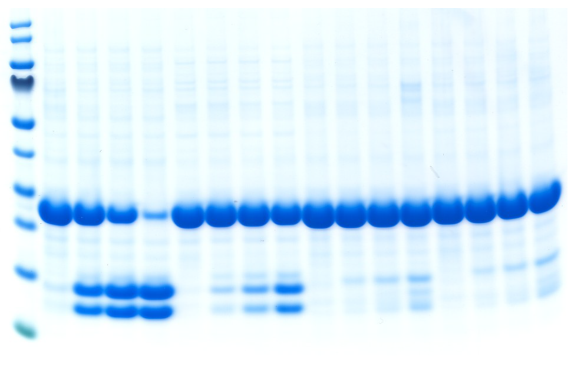

Figure 8C

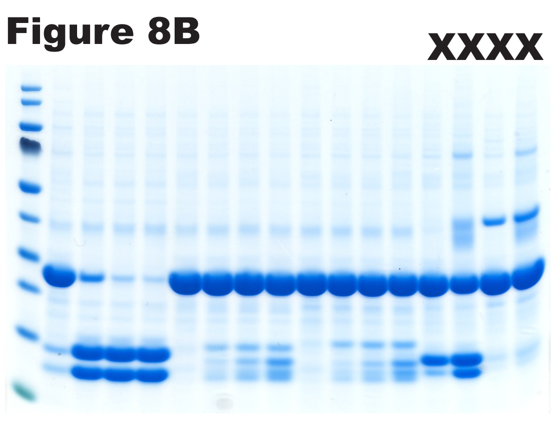

Figure 8D

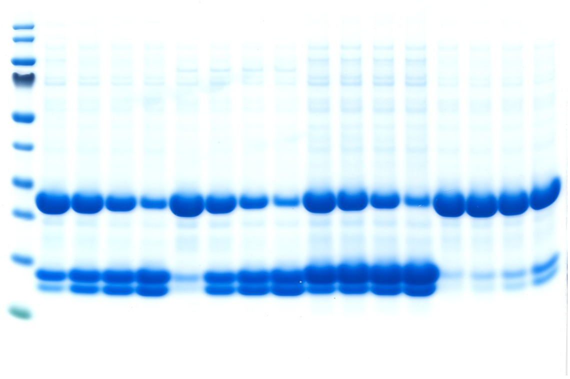

Figure 9A

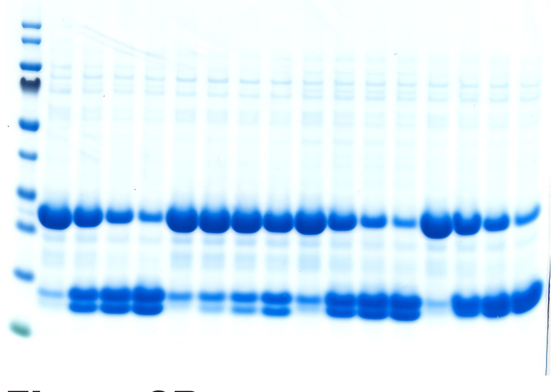

Figure 9B

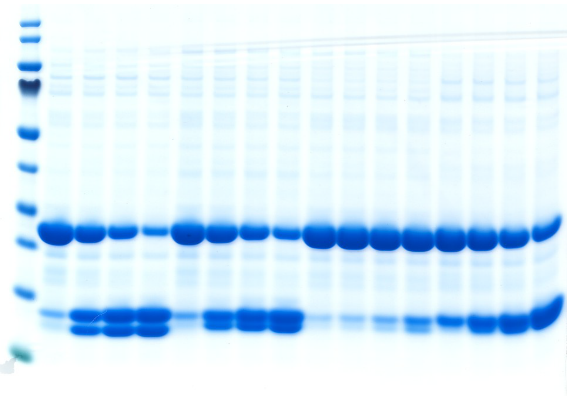

Figure 9C

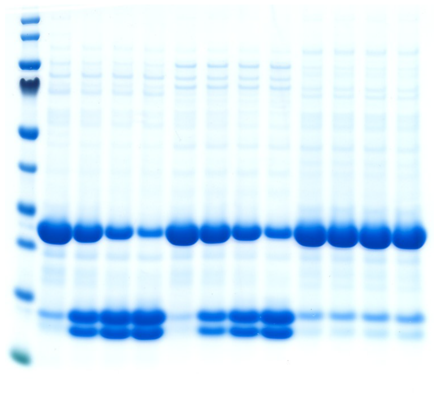

Figure 9D

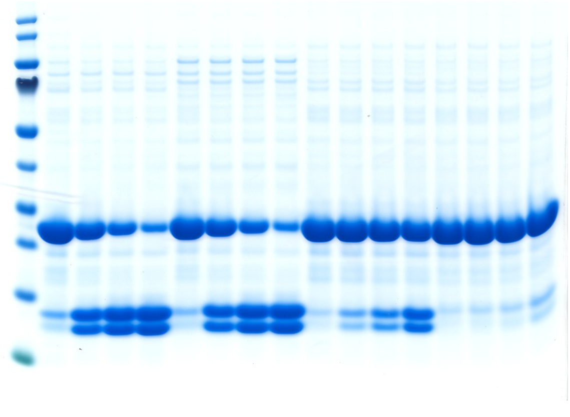

Figure 9E

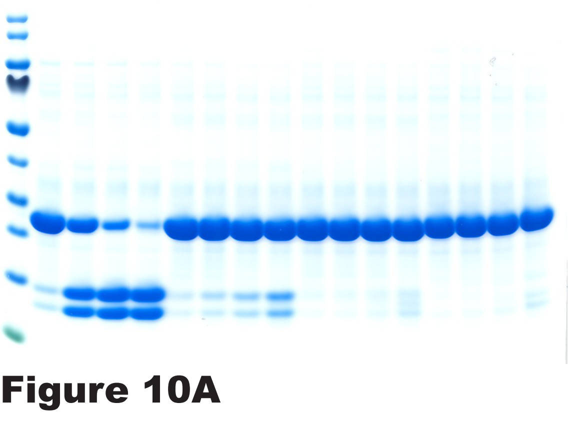

Figure 10A

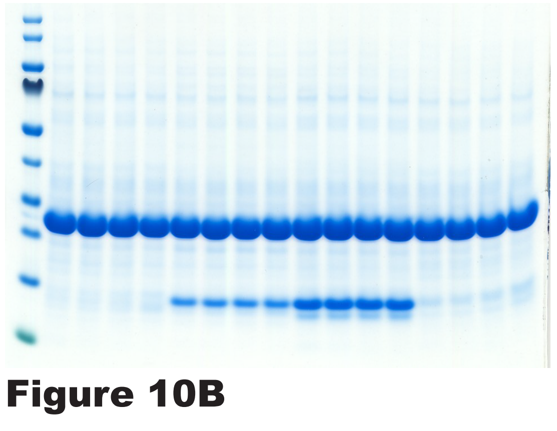

Figure 10B

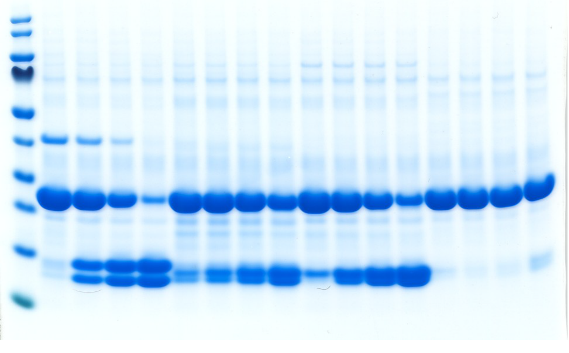

Figure 10C

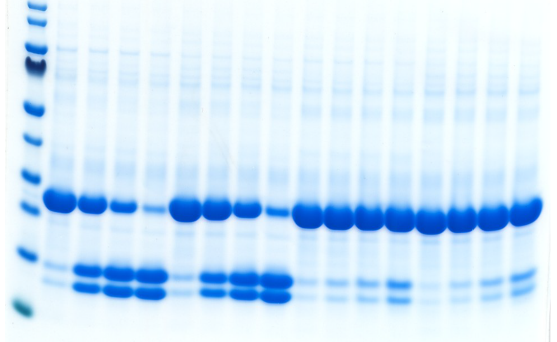

Figure 10D

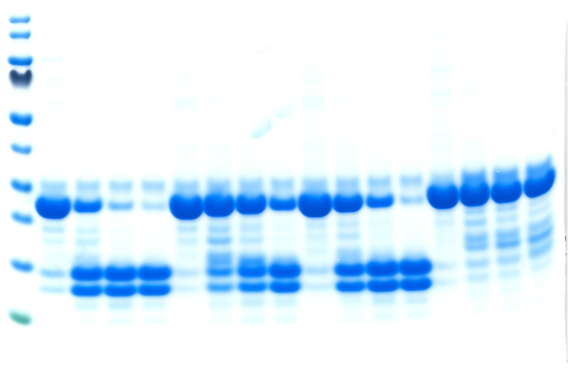

Figure 11A

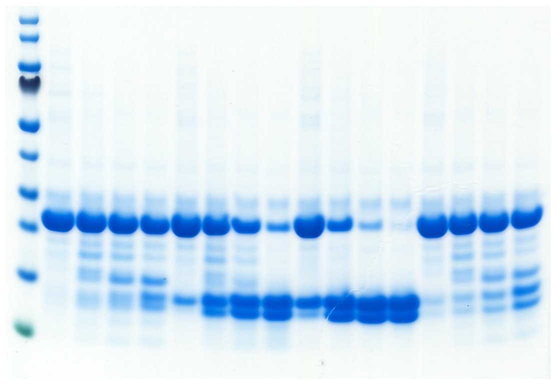

Figure 11B

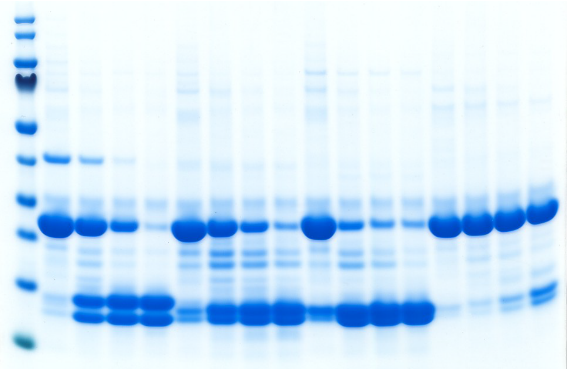

Figure 11C

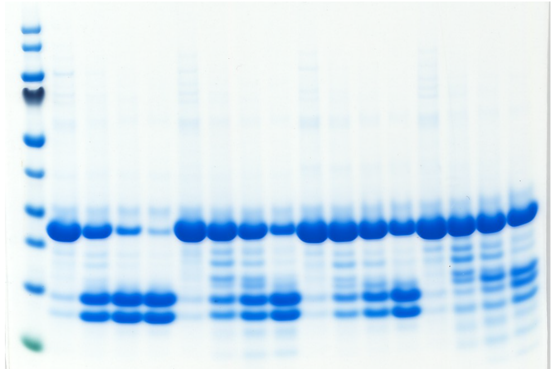

Figure 11D

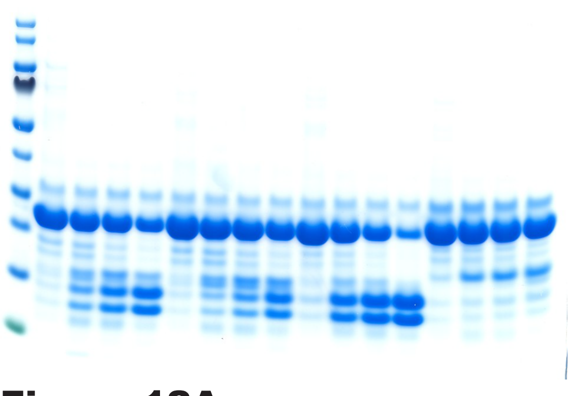

Figure 12A

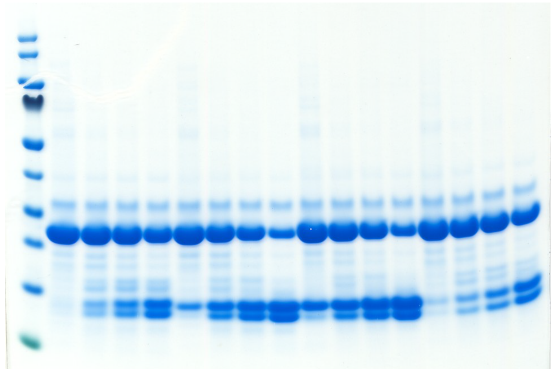

Figure 12B

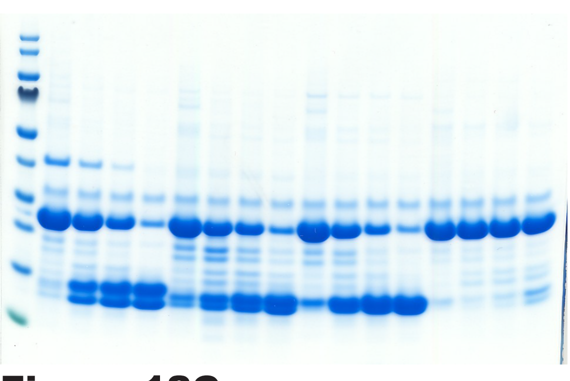

Figure 12C

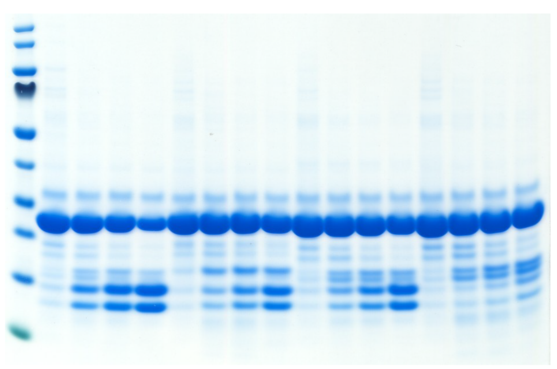

Figure 12D
